# Weak Electromagnetic Fields Accelerate Fusion of Myoblasts

**DOI:** 10.3390/ijms22094407

**Published:** 2021-04-23

**Authors:** Dana Adler, Zehavit Shapira, Shimon Weiss, Asher Shainberg, Abram Katz

**Affiliations:** 1 Faculty of Life Sciences, Bar Ilan University, Ramat Gan 52900, Israel; danadlerp@gmail.com (D.A.); asher.shainberg@gmail.com (A.S.); 2 Department of Physics, Bar Ilan University, Ramat Gan 52900, Israel; zehaviy@gmail.com (Z.S.); shiweiss@g.ucla.edu (S.W.); 3 Department of Chemistry and Biochemistry, University of California, Los Angeles, CA 90095, USA; 4 Åstrand Laboratory of Work Physiology, The Swedish School of Sport and Health Sciences, GIH, Box 5626, SE-114 86 Stockholm, Sweden

**Keywords:** creatine kinase, differentiation, fusion, myoblasts, myotubes, weak electromagnetic fields

## Abstract

Weak electromagnetic fields (WEF) alter Ca^2+^ handling in skeletal muscle myotubes. Owing to the involvement of Ca^2+^ in muscle development, we investigated whether WEF affects fusion of myoblasts in culture. Rat primary myoblast cultures were exposed to WEF (1.75 µT, 16 Hz) for up to six days. Under control conditions, cell fusion and creatine kinase (CK) activity increased in parallel and peaked at 4–6 days. WEF enhanced the extent of fusion after one and two days (by ~40%) vs. control, but not thereafter. Exposure to WEF also enhanced CK activity after two days (almost four-fold), but not afterwards. Incorporation of ^3^H-thymidine into DNA was enhanced by one-day exposure to WEF (~40%), indicating increased cell replication. Using the potentiometric fluorescent dye di-8-ANEPPS, we found that exposure of cells to 150 mM KCl resulted in depolarization of the cell membrane. However, prior exposure of cells to WEF for one day followed by addition of KCl resulted in hyperpolarization of the cell membrane. Acute exposure of cells to WEF also resulted in hyperpolarization of the cell membrane. Twenty-four hour incubation of myoblasts with gambogic acid, an inhibitor of the inward rectifying K^+^ channel 2.1 (K_ir_2.1), did not affect cell fusion, WEF-mediated acceleration of fusion or hyperpolarization. These data demonstrate that WEF accelerates fusion of myoblasts, resulting in myotube formation. The WEF effect is associated with hyperpolarization but WEF does not appear to mediate its effects on fusion by activating K_ir_2.1 channels.

## 1. Introduction

Living organisms are continuously exposed to weak electromagnetic fields (WEF) that can result in multiple biological consequences. For example, WEF can alter Ca^2+^ handling in various cell types, including cells of the immune system, stem cells, osteocytes, cardiomyocytes, and neurons [1,2,3,4,5]. Recently, we showed that WEF inhibits action potential and hypoxia-mediated increases in intracellular Ca^2+^ concentration ([Ca^2+^]_i_) and protects against muscle damage induced by hypoxia in rat primary skeletal muscle cultures [6]. Similar findings were subsequently observed in cultured cardiomyocytes [7].

That Ca^2+^ is involved in myogenesis in vitro was established 50 years [8]. Specifically, Ca^2+^ has been implicated in fusion and differentiation of myoblasts [8,9] and this has been subsequently confirmed in various laboratories [10,11,12,13,14,15,16,17]. It has been suggested that Ca^2+^ influx is indispensable for fusion and that, at least in human myoblasts, this occurs via T-type Ca^2+^ channels that open subsequent to membrane hyperpolarization [14]. The hyperpolarization appears to be dependent on activation of several different types of K^+^ channels [14]. Whether WEF affects muscle development, however, is not known. In view of our earlier findings that WEF altered Ca^2+^ handling [6], it appeared likely that muscle growth would also be affected by WEF. Therefore, we examined the effects of WEF on the fusion of skeletal muscle myoblasts in culture. The results demonstrate that WEF accelerates myoblast fusion, resulting in myotube formation.

## 2. Results

### 2.1. WEF Accelerates Fusion

**Figure 1:**
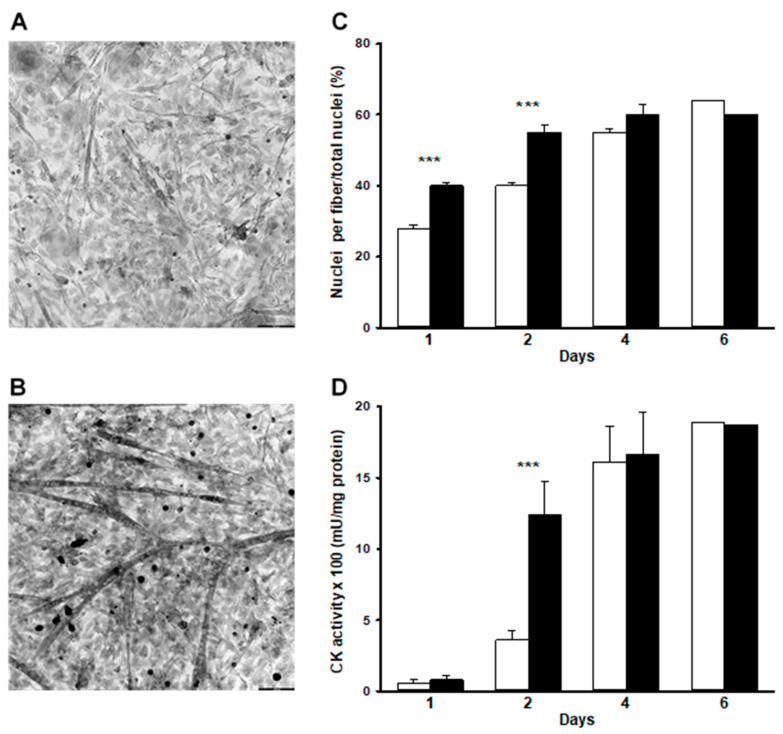
Effect of WEF on myoblast differentiation. Muscle cultures were either untreated (control) or exposed to WEF for up to six days. (**A**). Representative cell morphology using phase contrast microscopy for cells grown under control conditions for 1 day and (**B**). during exposure to WEF for one day (bar = 100 µm). (**C**). Presence of nuclei in myotubes over time in control (☐) and during exposure to WEF (■). Values are mean ± SE for 13–23 dishes (except for 6 days where only one dish was examined). (**D**). Creatine kinase activity over time in control (☐) and during exposure to WEF (■). Values are mean ± SE for 13–15 dishes (except for six days where only one dish was examined). *** *p* < 0.001 between groups at indicated day.

Fusion of myoblasts increased continuously, resulting in a value of ~60% of nuclei in myotubes by day 6 (Figure 1C), which is consistent with previous studies using this model [9]. CK activity increased in a parallel fashion (Figure 1D), which reflects gene activation during differentiation [18]. Exposure of cells to WEF enhanced the extent of fusion by ~40% after the first 2 d of treatment, but not thereafter (Figure 1A–C). WEF also resulted in increased CK activity after 2 d exposure to WEF, but not thereafter (Figure 1D).

### 2.2. Ca^2+^ Is Required for Fusion

**Figure 2:**
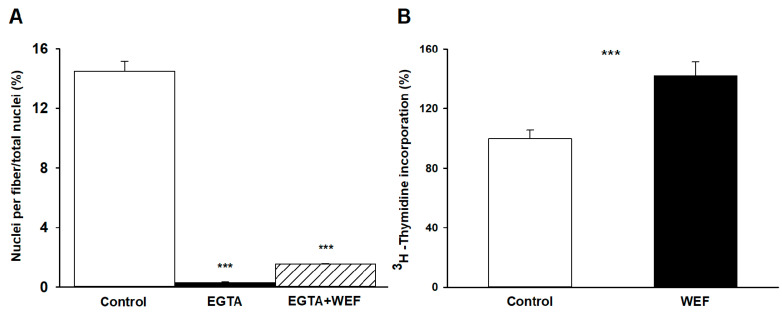
Effect of EGTA on myoblast differentiation and WEF on thymidine incorporation. (**A**) Presence of nuclei in myotubes in absence (Control) or presence of EGTA (1.8 mM), or EGTA + WEF for 24 h. In this series, experiments began 40 rather than 48 h after plating. Values are mean ± range for 3 dishes. *** *p* < 0.001 vs. Control. (**B**) Cells were grown with ^3^H-thymidine under control conditions for 24 h (☐) or exposed to WEF for 24 h (■). Values are mean ± SE for seven dishes. *** *p* < 0.001 between groups.

To examine if extracellular Ca^2+^ was required for WEF to exert its effects on myoblast fusion, cells were treated with EGTA. Addition of EGTA to culture medium prevented fusion of the cells (Figure 2A), which is consisted with earlier findings demonstrating that EGTA inhibits muscle cell fusion but does not affect viability [19]. Exposure of cells treated with EGTA to WEF did not enhance fusion, indicating that extracellular Ca^2+^ was required for fusion both in the absence and presence of WEF.

### 2.3. WEF Enhances Cell Replication

DNA synthesis, which reflects cell replication, occurs primarily during the initial 48 h of incubation, decreases sharply after 48 h, and does not require significant concentrations of extracellular Ca^2+^ [8]. We, therefore, examined whether WEF also affects this process by following the incorporation of ^3^H-thymidine into TCA-precipitates. Indeed, WEF significantly increased DNA synthesis, reflecting enhanced cell replication (Figure 2B).

### 2.4. WEF Causes Membrane Hyperpolarization

**Figure 3:**
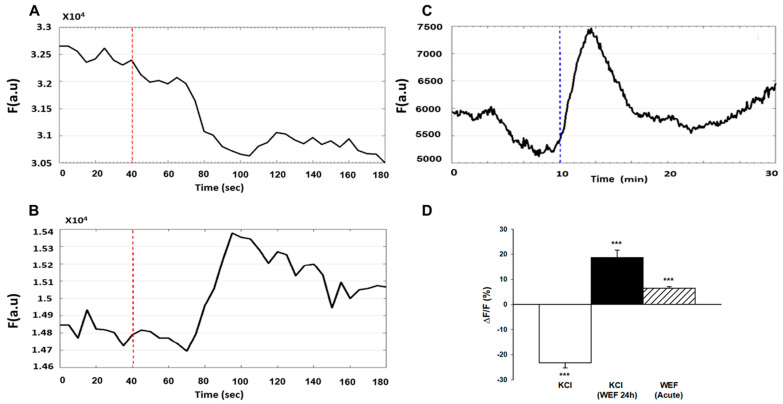
Effect of WEF on membrane potential of myotubes. Cells loaded with di-8-ANEPPS for 3 min were washed and followed for 3–30 min. (**A**). Representative trace of a control cell exposed to KCl (150 mM) at the time indicated (dashed line). Downward deflection represents depolarization. (**B**). Representative trace of cell exposed to WEF for 24 h, followed by interruption of ~10 min (for loading with di-8-ANEPPS and focusing in microscope), and then again exposed to WEF before addition of KCl at the time indicated (dashed line). Upward deflection represents hyperpolarization. (**C**). Representative trace of cell exposed to WEF for 24 h, followed by interruption of ~20 min (for loading with di-8-ANEPPS, focusing in microscope, and allowing for stabilization of baseline), before exposing to WEF as indicated (dashed line). (**D**). Mean ± SE values for 75 (KCl), 53 (KCl + WEF 24 h) and 7 (WEF acute) cells. *** *p* < 0.001 vs. 0.

Since membrane hyperpolarization has been implicated in myoblast fusion [13,14], we investigated whether WEF affected membrane potential. Earlier it was shown that di-8-ANEPPS fluorescence changed in response to changes in voltage across the cell membrane (decreased fluorescence reflects a decrease in membrane potential, and vice versa) [20]. As an increase in extracellular KCl causes membrane depolarization, we first confirmed that di-8-ANEPPS documents such an event under our conditions of study. Indeed, by using di-8-ANEPPS, a membrane potential increase (i.e., depolarization) was observed in response to administration of KCl (Figure 3A). However, in the presence of chronic exposure to WEF, addition of KCl resulted in hyperpolarization (Figure 3B). To examine the direct effects of WEF on membrane potential, cells that were exposed to WEF for 24 h were loaded with di-8-ANEPPS for 3 min. After a stabilization period of 10 min, cells were again exposed to WEF for 20 min. WEF induced hyperpolarization that became apparent within several min (Figure 3C). The mean values for these experiments are summarized in Figure 3D.

### 2.5. WEF Does Not Activate K_ir_2.1 Channels

**Figure 4:**
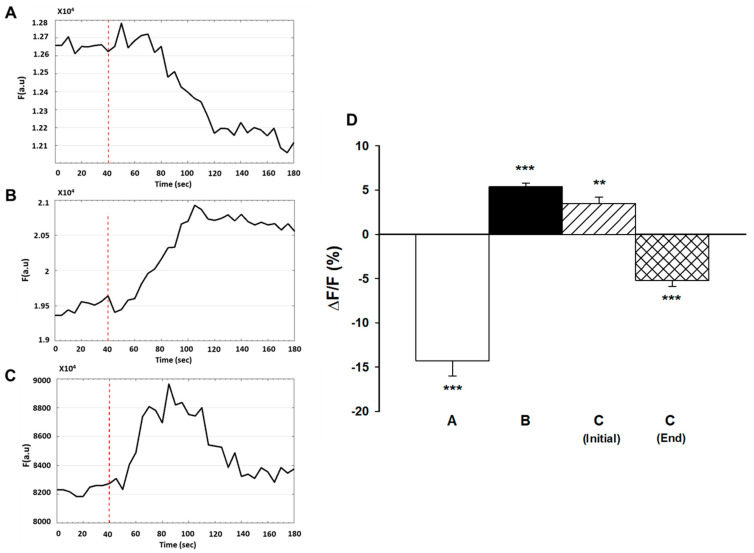
Effect of gambogic acid (GA) on membrane potential of myotubes induced by KCl. Cells were exposed to 200 nM GA and loaded with di-8-ANEPPS as described in legend to Figure 4. (**A**). Representative trace of a cell treated with GA for 24 h and exposed to KCl at the time indicated (dashed line). (**B**). Representative trace of a cell exposed to WEF for 24 h and subsequently to GA and WEF for 60 min before administration of KCl at the time indicated (dashed line). (**C**). Representative trace of a cell exposed to WEF and GA for 24 h before administration of KCl at the time indicated (dashed line). Note the blunted and transient response. (**D**). Mean ± SE values for cells in trace A (*n* = 24), trace B (*n* = 27), and C (*n* = 6, initial indicates increase; end indicates decrease from peak). ** *p* < 0.01; *** *p* < 0.001 vs. 0.

**Figure 5:**
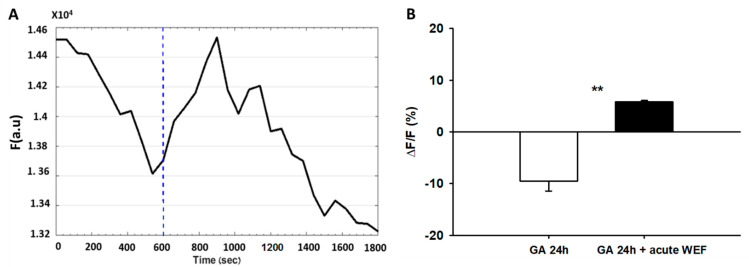
Gambogic acid (GA) does not block WEF-mediated hyperpolarization. (**A**). Representative trace of a cell exposed to GA for 24 h and subsequently exposed to WEF as indicated (dashed line). (**B**). Mean ± SE values for three cells. ** *p* < 0.01 between groups.

Previous studies demonstrated that myoblast fusion and differentiation were dependent on hyperpolarization that was due to increased expression and activity of K_ir_2.1 channels [12,13,14,21]. To investigate the role of K_ir_2.1 channels in WEF-dependent hyperpolarization, GA, a potent inhibitor of the K_ir_2.1 channel (inhibitory constant, IC_50_ of 27 nM), was used [22,23]. Exposure of cells to 200 nM GA for 25 h did not affect myoblast fusion either in the absence or presence of 24 h exposure to WEF (data not shown). To assess the bioactivity of GA, experiments with membrane potential were also performed. Prolonged exposure to GA (25 h) did not affect KCl-mediated membrane depolarization (Figure 4A). As KCl-mediated depolarization likely occurs due to slowing of K^+^ efflux via leak channels [24], these results indicate that GA does not affect the K^+^ leak channels. Indeed, under the conditions studied, GA does not inhibit other K^+^ channels, including K_v_2.1, hERG or K_ir_1.1 channels [23]. Following 24 h exposure to WEF, addition of KCl (while cells were still being exposed to WEF) resulted in hyperpolarization (as in Figure 3B) and this, too, was not affected by 1 h exposure to GA (Figure 4B). However, following 25 h exposure to GA and 24 h exposure to WEF, addition of KCl resulted in a transient, blunted degree of hyperpolarization (Figure 4C), indicating that the drug was biologically active. Data from this series are summarized in Figure 4D. Finally, prolonged exposure to GA did not block hyperpolarization induced by acute exposure to WEF (Figure 5). This suggests that WEF-mediated hyperpolarization does not derive from activated K_ir_2.1 channels.

## 3. Discussion

Many epidemiological associations between electromagnetic fields and disease have been reported, but the causality of the relationships is generally not supported by knowledge of known mechanisms [25]. Still there are numerous reports of positive effects of electromagnetic fields on musculo-skeletal disorders as reviewed earlier [26]. More recently, beneficial effects of electric fields on wound healing and tissue regeneration have been documented [27]. Finally, there is now considerable evidence for synergistic effects of pharmacological compounds and WEF on neural functions [28]. It follows that understanding the mechanisms whereby WEF affects biological functions is paramount for optimal application of electric fields in human health.

In the present study we investigated the effects of WEF on muscle growth and examined potential mechanisms of action. The major findings are that: (1) WEF accelerates fusion of myoblasts; (2) WEF induces membrane hyperpolarization and cell proliferation; and (3) membrane hyperpolarization via K_ir_2.1 channel is not a prerequisite for WEF to exert its effect during the initial stages of muscle growth.

The finding that WEF increased the extent of fusion during the initial 48 h of treatment, but not thereafter, suggests that it accelerated the activity of an inherent process, rather than activated a separate mechanism of action. Often, prior to fusion, myoblasts replicate and migrate [29]. Subsequently, fusion involves cell adhesion, hyperpolarization and activation of signal transduction [13,14,30]. Cell division occurs initially and upon reaching an optimal cell density fusion follows [29]. Thus, WEF may incur its positive effects on fusion by accelerating any of the above processes. We examined the effects of WEF on both cell replication and membrane hyperpolarization. Indeed both were increased and could be involved in the enhancement of fusion during the initial stages of myogenesis. In this context, it is of interest that the extent of cell replication and fusion were both ~40%.

We hypothesized that WEF exerted its effects on fusion by enhancing K^+^ efflux via K_ir_2.1 channels, which will result in hyperpolarization. This was based on the observation that studies on human myoblasts demonstrated that there is a rapid increase in the expression of K_ir_2.1 during the initial 24 h of culture that is associated with hyperpolarization, followed by fusion [12,13,14,21]. Blocking the K_ir_2.1 current inhibited fusion [14,21]. The fact that exposure to WEF caused hyperpolarization within several minutes suggested that the K_ir_2.1 channel explanation was plausible. However, usage of GA under conditions that should fully inhibit function of the K_ir_2.1 did not affect the fusion induced by WEF. Therefore, the results did not support involvement of the latter channels in the WEF-mediated enhancement of fusion. Our results, however, are consistent with an important role of hyperpolarization in WEF-mediated enhancement of fusion of myoblasts.

Previously we showed that WEF abolished action-potential mediated Ca^2+^ transients, but we could not determine whether this occurred because of inhibition of dihydropyridine receptors or inhibition of membrane depolarization [6]. The observation that KCl-mediated depolarization was blocked (and even reversed) by prolonged exposure to WEF in the present study indicates that WEF inhibits action-potential mediated Ca^2+^ transients by inhibiting membrane depolarization. Nevertheless, the link between muscle growth, WEF and Ca^2+^ handling is not fully understood. Significant extracellular Ca^2+^ (1400 µM) is requisite for fusion of myoblasts [8], as well as to observe the enhancing effect of WEF on fusion (present findings). In contrast, a number of metabolic processes associated with growth/replication, including DNA, RNA and protein synthesis are essentially normal in medium containing a Ca^2+^ concentration of only 70 µM [8]. WEF also enhanced DNA synthesis/cell replication in the presence of high extracellular Ca^2+^ (1.8 mM). However, it is not known whether the WEF effect on replication also occurs at a low extracellular Ca^2+^ concentration.

In conclusion, WEF enhances myoblast replication and fusion. The effect of WEF on fusion requires high extracellular Ca^2+^, is associated with hyperpolarization, but WEF does not involve functional K_ir_2.1 channels. Considering the number of positive applications of electromagnetic fields in human health [26,27], WEF may prove useful in accelerating skeletal muscle regeneration following acute trauma.

## 4. Materials and Methods

### 4.1. Animals and Materials

Sprague-Dawley rat pups (1–2 days of age) were purchased from Envigo, Jerusalem, Israel. The pups were killed by decapitation and skeletal muscles were dissected from the thigh for subsequent preparation (see below). All experiments were conducted according to the guidelines of the National Institutes of Health for the care and use of laboratory animals and approved by the institutional review board of Bar-Ilan University (Ethical number 98-12-2014).

All chemicals were from Sigma-Aldrich unless stated otherwise. ^3^H-thymidine was from Perkin-Elmer (Waltham, MA, USA). Polyclonal antibody against an extracellular epitope of the inward rectifying K^+^ channel 2.1 (K_ir_2.1) was purchased from Alomone Labs (#APC-159, Jerusalem, Israel). Gambogic acid (GA) was purchased from Abcam (ab145183, Cambridge, UK). The potentiometric fluorescent dye, di-8-ANEPPS, was purchased from Biotium (#61014, Fremont, CA, USA).

### 4.2. Experimental

We studied primary rat muscle cell cultures, a model that is well established in our laboratory. These cells are more likely to exhibit properties resembling those in vivo, as opposed to muscle cell lines that may display variations in genotype and phenotype during serial passages. Muscle cultures were prepared as described previously [9]. Briefly, skeletal muscle was removed under sterile conditions and washed three times with phosphate-buffered saline (PBS) to remove excess blood. PBS consisted of (in mM) the following: NaCl 135, KCl 3.7, Na_2_HPO_4_ 10, KH_2_PO_4_ 1.8, MgCl_2_ 0.5, CaCl_2_ 0.9, yielding a pH of 7.3. Muscles were minced finely and gently agitated in PBS containing 0.25% trypsin, for a few cycles of 10 min each, which resulted in the release of single cells. The suspension was then centrifuged for 5 min at 500× *g* at room temperature. The supernatant was discarded and cells were re-suspended in Dulbecco’s Modified Eagle Medium (DMEM) containing 25 mM glucose and supplemented with 10% heat-inactivated horse serum and 2% chick embryo extract. The cell suspension was diluted in the same medium to 1.2 × 10^6^ cells/mL and 1.5 mL of cells was then plated in 35 mm collagen/gelatin coated plastic culture dishes or cover glasses. Cultures were incubated in a humidified atmosphere of air with 5% CO_2_ at 37 °C. Growth medium was changed after 24 h. Thereafter medium was changed every 72 h. Interventions began 48 h after plating. Thus, all treatment durations reported refer to those after the initial 48 h after plating, unless stated otherwise. Cells were exposed to WEF (1.75 µT, 16 Hz, 5 V), generated by a stimulator connected to a copper wire coil with a single wrapping around the culture dish in the incubator (or inverted microscope—see below) for the durations indicated (see Results). This field strength and frequency was chosen since they were found to be optimal for studying the effect of WEF on Ca^2+^ handling [6]. Additional details are available elsewhere [6]. In one series of experiments 1.8 mM EGTA was added to the culture dishes to chelate extracellular Ca^2+^. In another series, KCl (final concentration 150 mM) was added to induce membrane depolarization. The extracellular cell recording solution was composed of (in mM): 150 NaCl, 5 KCl, 2 CaCl_2_, 1 MgCl_2_, 5 HEPES, 20 glucose, and pH was adjusted to 7.4. Depolarization cell recording solution (high K^+^ concentration) was injected at a constant rate and consisted of (in mM): 150 KCl, 2 CaCl_2_, 1 MgCl_2_, 5 HEPES, 25 glucose, 2 NaCl, and pH was adjusted to 7.4. The measured osmolarity for the two solutions ranged from 322–328 mOsm/L and was adjusted with sucrose.

To assess membrane potential changes resulting from exposure to WEF, we used di-8-ANEPPS, a voltage sensitive fluorescent dye. The microscope set-up for the measurement of changes in membrane potential experiments consisted of an Olympus IX83 inverted microscope equipped with a LED lamp (SPECTRA X, Lumencor Inc., Beaverton, OR, USA). The culture was excited at 480 nm and the emitted light of the di-8-ANEPPS dye was collected by a 40× objective lens and passed through a 505 nm long-pass dichroic mirror and a 510 nm longpass emission filter. Imaging was performed with an electron multiply charge coupled device (EMCCD, Andor iXon, South Windsor, CT, USA). Cells were loaded with di-8-ANEPPS (5 µM in 1% DMSO and medium) for 3 min and washed three times. Thereafter, cells were treated as indicated (see Results). Frames were captured every 5 s for 3 min following interventions (for baseline experiments, frames were captured every 5 s for 30 min). Results are derived from three separate cell culture preparations generated at intervals of at least 3 separate days, with three plates for each experiment, unless stated otherwise. For obtaining images of several cells in the same plate, 2–4 cells were followed from each plate on the same frame recording under the same conditions. Additionally, three different regions were sampled from each cell to ensure that the same trajectories were obtained.

Changes in the fluorescence of di-8-ANEPPS upon chemical polarization (in percent) were calculated by subtracting the baseline mean intensity in the relevant region of interest from maximal measured mean intensity of the same region ((ΔF/F) × 100). We established that changes in fluorescence corresponded to changes in membrane potential by performing patch clamp experiments on primary cultured neurons. Holding potentials varied between −60 to +60 mV using 20 mV steps (step duration was 200 ms over a period of 42 s). Under these conditions di-8-ANEPPS exhibited a voltage sensitivity of 5 + 1% ((ΔF/F) × 100) per 120 mV, with a response time of 10 ms (data not shown). Earlier studies have established a di-8-ANNEPS voltage sensitivity of ~15% per 100 mV in skeletal muscle cells [31]. Results that were exceptional were excluded and not considered in statistical calculations. The trajectories graphs presented are representative results and not means.

### 4.3. Analyses

For measuring creatine kinase (CK) activity, cells were washed twice with PBS and then scraped in 1 mL of ice cold PBS. The mixture was sonicated (10 s) on ice and centrifuged at 4 °C for 10 min at 3000× *g*. Twenty-five µL of supernatant were assayed for CK with a spectrophotometric method following the production of NADPH using a CK/NAC kit (Thermo Scientific, TR 14010, Waltham, MA, USA). Protein in supernatant was analyzed with the Bio-Rad assay (Hercules, CA, USA). To assess cell replication, cells were incubated with ^3^H-thymidine (1 µCi/mL medium) for 24 h. Thereafter, cells were washed 5 times with ice-cold PBS, scraped in 0.5 mL of PBS and sonicated (10 s). A total of 0.5 mL of ice-cold 10% TCA was added to the homogenate and the mixture was incubated on ice for 15 min, followed by 10 min of centrifugation at 3000× *g*. The supernatant was discarded and soluene was added to solubilize the pellet. The latter was transferred to 4 mL of scintillation cocktail (Ultima Gold, Merck, Germany) and counted for radioactivity.

Cell fusion was assessed by counting the number of nuclei (≥3, stained with Giemsa) within myotubes divided by the total number of nuclei counted in the microscope field (objectives of 40× or 20×) and the values were expressed in % of total. Nine different fields were chosen randomly for counting and the mean was calculated as representative for a dish.

### 4.4. Statistical

Values are presented as mean ± SE, unless stated otherwise. Statistically significant differences (*p* < 0.05) between two means were calculated with unpaired *t*-tests and for more than two means (Figure 2) with a one-way ANOVA, followed by an LSD post-hoc test. Use of ANOVA was based on the observation that skewness statistics indicated a normal distribution. Changes within a cell were calculated with paired *t*-tests (Figure 3 and Figure 4). The results are derived from ≥3 separate experiments (i.e., three separate cell preparations on three separate days) unless stated otherwise.

## Data Availability

Data are available to readers upon reasonable request.
